# Mild autonomous cortisol secretion (MACS) is associated with increased mortality in patients with adrenal adenomas only in the presence of type 2 diabetes: a retrospective cohort study

**DOI:** 10.1186/s12933-026-03360-4

**Published:** 2026-09-22

**Authors:** Luise Bellach, Sarah Hofer-Zeni, Dorota Sluková, Dorothea Kalender, Clemens Zimmermann, Naima Michler, Liliana Gard, Sabina Baumgartner-Parzer, Rodrig Marculescu, Greisa Vila, Peter Wolf, Alexandra Kautzky-Willer, Michael Leutner

**Affiliations:** 1 Department of Internal Medicine III, Division of Endocrinology and Metabolism, Medical University of Vienna, Vienna, Austria; 2 Department of Laboratory Medicine, Medical University of Vienna, Vienna, Austria

**Keywords:** Diabetes mellitus, hypercortisolism, MACS, adrenal adenomas, mortality, cardiovascular disease

## Abstract

**Background:**

Hypercortisolism and type 2 diabetes mellitus (T2D) independently contribute to an increased burden of cardiometabolic morbidity and mortality. However, data on the interaction of hypercortisolism and T2D are scarce—thus, we aimed to assess the cortisol-associated morbidity and mortality in the context of T2D and adrenal mild autonomous cortisol secretion (MACS).

**Methods:**

This is a retrospective cohort study including 668 patients with adrenal adenomas, who were visiting the endocrine outpatient clinic at the Vienna General Hospital between 01.01.2000 to 31.12.2022. Of those, 151 were diagnosed with T2D at the time of their 1 mg dexamethasone suppression test (DST), other diabetes types were excluded. MACS was defined as a post 1 mg DST cortisol of > 1.8 µg/dL in the absence of Cushing’s stigmata and after biochemical exclusion of ACTH-dependency. The database was linked to the governmental death registry and patients alive after 01.12.2024 were censored. Kaplan Meier curves and cox proportional hazard models were analyzed via RStudio.

**Results:**

Patients with T2D had a higher post 1 mg DST cortisol level and were more often diagnosed with hypertension, dyslipidemia and cardiovascular disease (CVD) compared to those without T2D. Compared to patients with non-functioning adrenal adenomas (NFAA) without T2D, MACS was strongly linked to increased mortality only in T2D (HR 2.51 [CI95% 1.41–4.48], *p* < 0.01) but not in those without T2D, even after adjustment for age, sex, BMI, smoking, hypertension, dyslipidemia and CVD at baseline. The T2D cohort with MACS had higher HbA1c and a 3-fold elevation in CVD prevalence compared to those without hypercortisolism. They did not differ according to age, sex or other cardiometabolic risk parameters (dyslipidemia, hypertension, BMI).

**Conclusions:**

Among patients with adrenal adenomas, MACS is related to increased CVD prevalence and is associated with mortality in patients with T2D. These findings highlight the need for confirmatory prospective studies incorporating larger sample sizes to further assess the importance of earlier identification and treatment of MACS in patients with T2D.

**Graphical abstract:**

**Figure:**
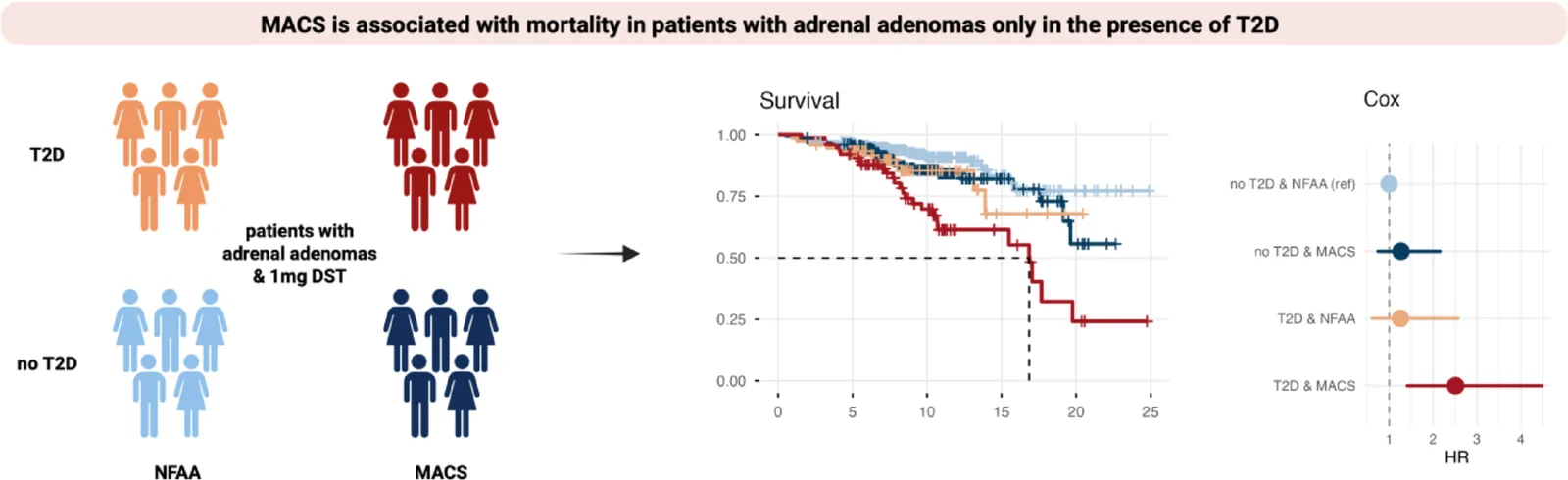

**Supplementary Information:**

The online version contains supplementary material available at https://doi.org/10.1186/s12933-026-03360-4.

## Research highlights

**What is currently known about this topic?**

Both hypercortisolism and diabetes increase cardiometabolic morbidity and mortality.

Specific treatment may be considered for cases without Cushing’s stigmata.

**What is the key research question?**

What is the effect of diabetes in adrenal mild autonomous cortisol secretion (MACS) on morbidity and mortality?

**What is new?**

Among patients with adrenal adenomas, MACS increases mortality only in patients with type 2 diabetes.

MACS is linked to a higher HbA1c in patients with diabetes and adrenal adenomas.

Diabetes and MACS are linked to a 3-fold increase in cardiovascular disease prevalence in patients with adrenal adenomas.

**How might this study influence clinical practice?**

Our findings support future studies investigating the effects of cortisol lowering treatment in the presence of diabetes and MACS.

## Background

In patients with adrenal adenomas, mild autonomous cortisol secretion (MACS) represents a common form of subclinical hypercortisolism and is reported in 20–50% of cases. The term MACS was only recently introduced by the European Society of Endocrinology (ESE) in [1] due to mounting data on increased mortality even in mild hypercortisolism [2] and encompasses both patients who fail to suppress cortisol levels ≤ 1.8 µg/dL in an overnight 1 mg dexamethasone suppression test (DST) and who, at the same time, do not exhibit clinical symptoms of overt Cushing’s syndrome.

This is especially relevant in the context of glycemic control as chronic exposure to excess cortisol is closely linked to insulin resistance and the development of type 2 diabetes mellitus (T2D) [3–5]. Accordingly, a recent meta-analysis reported a higher prevalence of comorbidities such as diabetes mellitus, hypertension and dyslipidemia in patients diagnosed with MACS than in those with non-functioning adrenal adenomas (NFAA) [6]. Vice versa, Ferrari et al. recently demonstrated in a meta-analysis that up to 14% of patients with diabetes mellitus present with an abnormal DST [7]. Furthermore, Favero et al. demonstrated an association between higher post 1 mg DST cortisol levels and diabetes prevalence even among patients with non-functioning adrenal adenomas and suggested that a lower DST cut-off may improve the identification of patients with clinically relevant cortisol excess [8].

The association between hypercortisolism and cardiovascular diseases (CVD) has been described repeatedly [9]. This is also observed in patients with adrenal adenomas. Di Dalmazi et al. and Morelli et al. reported higher prevalence of CVD with increasing degrees of cortisol secretion in patients with adrenal adenomas [10, 11]. Longitudinal data further indicate that higher post DST cortisol levels are associated with an increased incidence of cardiovascular events in patients with adrenal adenomas [12]. This association may partly be mediated by the effect of cortisol on established risk factors, including arterial hypertension (aHTN), dyslipidemia and impaired glucose metabolism [6, 13, 14]. In particular, cortisol is a potent driver of gluconeogenesis, impairs insulin secretion, and decreases insulin-dependent glucose uptake [3, 4, 13]. Consistently, in a clinical study Chen et al. observed lower postprandial insulin secretion in patients with MACS than in those with non-functioning adrenal adenomas [15]. Thus, T2D may represent an important modifier of the prognostic impact of adrenal cortisol excess. However, data regarding the impact of adrenal hypercortisolism on mortality in patients with T2D remains scarce.

According to new guidelines, treatment should also be considered in MACS dependent on severity or progression of cortisol-related comorbidities such as T2D, aHTN, dyslipidemia or osteoporosis [1]. However, the prognostic relevance of specific comorbidities, as well as their role in guiding treatment decisions, has not yet been fully characterized in detail.

With MACS only recently added to the ESE guidelines for adrenal adenomas and considering the link between hypercortisolism, T2D and adverse outcomes, the present study aims to identify relevance of T2D in patients with adrenal adenomas depending on presence of hypercortisolism (MACS) regarding cortisol-associated morbidity and mortality.

## Methods

**Fig. 1:**
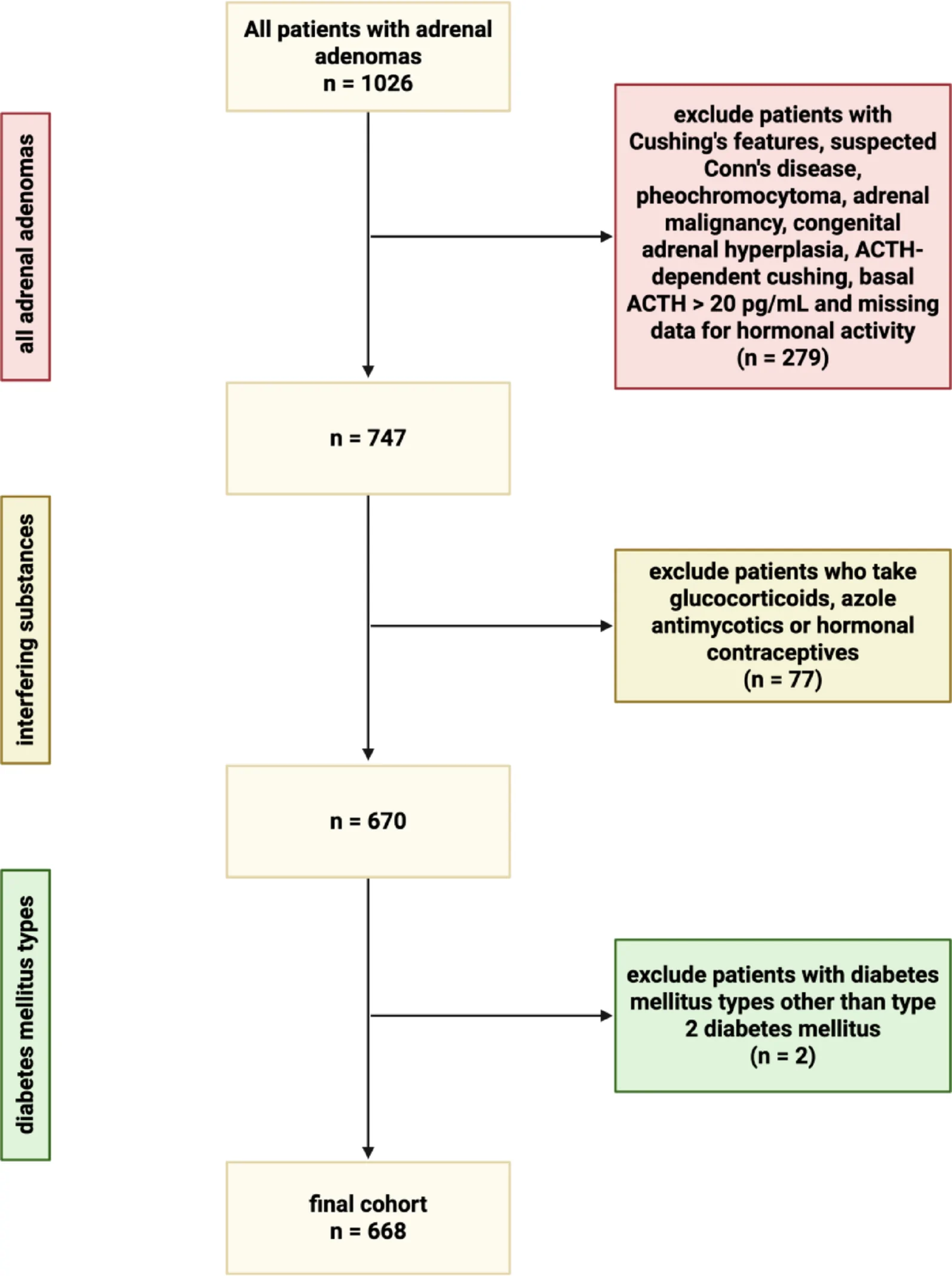
Flow chart illustrating patient selection. Created in BioRender. Bellach (2026) https://BioRender.com/icd1cje

This is a retrospective cohort study assessing the disease burden and mortality of patients with adrenal adenomas and type 2 diabetes mellitus. Adult patients with adrenal adenomas presenting for a diagnostic workup testing for adrenal hypercortisolism via a 1 mg DST at the outpatient clinic of the Department of Endocrinology and Metabolism of the Medical University of Vienna between 01.01.2000 and 31.12.2022 were included. A 1 mg dexamethasone suppression test was performed in all patients: patients were instructed to take 1 mg of dexamethasone orally at 23:00 and, the following day, cortisol levels were assessed at 09:00 am. According to the latest guidelines [1], patients with a post 1 mg DST serum cortisol level of over 1.8 µg/dL were categorized as having adrenal hypercortisolism (MACS) – patients with 1 mg DST cortisol levels of 1.8 µg/dL or below were categorized as NFAA. A hormone screening to rule out ACTH-dependency and other adrenal hormone production was performed in all patients. Overall, 1026 patients were screened. The inclusion criteria were a benign adrenal adenoma, a documented post 1 mg DST cortisol value and biochemical exclusion of ACTH independency. Documented Cushing features (extracted from medical records of the endocrine outpatient clinic), Conn’s disease, pheochromocytoma, congenital adrenal hyperplasia, malignant adrenal masses or ACTH-dependent states of hypercortisolism and patients taking any cortisol-interfering drugs (e.g. exogenous cortisol preparations, azole antimycotics and hormonal contraceptives) were excluded from the analysis. Other than azole antimycotics, no other CYP3A4-interacting drugs were considered in this study. Patients with documented basal ACTH values of over 20 pg/mL were excluded [16]. Also, patients with missing data regarding hormonal activity were excluded. Furthermore, diabetes types other than T2D were excluded from the analysis, the cohort without T2D was not further divided according to prediabetes. The filtering process is visualized in Fig. 1. Data were retrospectively collected from the patients’ electronic charts and documented in a database. Presence of T2D (defined as documented disease, use of antidiabetic drugs or per laboratory parameters according to the ADA 2026 guidelines [17]) and other cortisol-related comorbidities like CVD (defined as the documented presence of either coronary artery disease, cerebral artery disease or peripheral artery disease), dyslipidemia (defined as documented disease or use of lipid-lowering drugs), aHTN (defined as documented disease or use of antihypertensive drugs) and osteoporosis (defined as documented disease or use of antiresorptive drugs) were documented in a dichotomous manner from the medical health records. The first documented 1 mg DST constituted as baseline, aside from basal ACTH and DHEAS levels, no further hormonal assessments (follow-up 1 mg DST, dexamethasone concentrations, low-dose DST, urinary free cortisol, late-night salivary cortisol) were analyzed in this study. Cortisol levels were measured by the Elecsys competitive electroluminescence immunoassay (Roche, Mannheim Germany), which is traceable to isotope-dilution mass spectrometry during the entire study period. Measurements were performed on the Modular E170, Cobas e602 and Cobas e801 platforms. Inter-assay CV was < 10.1% and intra-assay CV was < 5.4% during the entire study period. Calibration and quality control procedures were performed according to manufacturer’s instructions and to the ISO 15,189 norm, including continuous participation in multiple external quality control programs.

### Statistical analysis

This analysis was conducted using RStudio (Version “Pacific Dogwood”) on R (Version 4.5.3). The packages used for the general statistical analyses were *tidyverse*, *rio*, *here*, *stats*, and *rstatix*. Missing data for BMI were imputed using the mice algorithm [18] to fit the successive models in the full cohort. Survival rates according to T2D in the context of MACS were the primary outcome parameter. P-value adjustment was performed via Benjamini-Hochberg’s method.

### Baseline characteristics

Data were displayed via count and percentage for categorical variables, respective testing for between-group differences was performed via chi2 test of independence and Fisher's exact test. For metric data, median and interquartile range was used with testing performed via Kruskal Wallis test and Wilcoxon rank-sum test.

### Survival analysis

For the survival analysis, we linked our data set to the governmental death registry provided by *Statistik Austria* [19] with a follow-up time of up to 01.12.2024. The time to event was defined retrospectively as time from first hormonal diagnostic testing of the adrenal adenoma (i.e. 1 mg DST) to time of death. Patients alive after 01.12.2024 were censored at that date. To assess the impact of T2D in the context of MACS on mortality in patients with adrenal adenomas, a Kaplan-Meier analysis compared the 3 different exposure combinations (no T2D & MACS (*n* = 201), T2D & NFAA (*n* = 75), and T2D & MACS (*n* = 76)) against a double-negative adrenal adenoma control cohort (no T2D & NFAA (*n* = 316)). The analysis was performed using the package *survminer*. Subsequently, successive cox proportional hazards models were performed via the *survival* package to account for sex, age, and cardiovascular risk modifiers like smoking status, BMI, aHTN, dyslipidemia and CVD. Sensitivity analyses of the full models excluding all patients with baseline CVD and correcting for adrenalectomy were performed. Furthermore, main-effects and interaction cox proportional hazards models were applied according to T2D and MACS categories.

## Results

### Baseline characteristics

In our cohort of 668 patients with adrenal adenomas 277 were categorized as MACS and 391 as NFAA – accordingly, patients with MACS had lower baseline ACTH and DHEAS values compared to NFAA (7 vs. 13 pg/mL and 0.53 vs. 0.85 µg/mL, respectively, *p* < 0.01, Table S1). 151 had a diagnosis of T2D at first visit. Patients with T2D were significantly older (63 vs. 58 years, *p* < 0.001), were less often female (55.0 vs. 65.6%, *p* < 0.05) and had a higher BMI (29.7 vs. 27.2 kg/m^2^, *p* < 0.001) compared to those without T2D. Also, they had higher median post 1 mg DST cortisol levels (1.9 vs. 1.5 µg/dL, *p* < 0.01) and exhibited higher rates of cardiometabolic comorbidities such as dyslipidemia (62.9 vs. 34.4%, *p* < 0.001), aHTN (85.4 vs. 61.1%, *p* < 0.001) and CVD (27.2 vs. 8.1%, *p* < 0.001) compared to those without T2D. There were no significant differences regarding osteoporosis rates (Table 1). The descriptive data regarding cardiometabolic drugs can be seen in *Table S2.*

In T2D- and MACS-specific subgroups the 4 cohorts (no T2D & NFAA, no T2D & MACS, T2D & NFAA, T2D & MACS) differed significantly in age, BMI, post 1 mg DST cortisol levels as well as in prevalence rates in aHTN, dyslipidemia, CVD (*p* < 0.001), osteoporosis (*p* < 0.01) and in sex (*p* < 0.05, Table S3). Regarding between-group differences, the cohorts differed predominantly according to T2D status (Table 1, Table S3). Within the cohort without T2D, patients with MACS were older (60 vs. 56 years, p < 0.05), had a lower BMI (26.2 vs. 28.2 kg/m^2^, *p* < 0.05) and had higher osteoporosis (9.5 vs. 2.5%, *p* < 0.05) prevalences. Importantly, patients with T2D did not differ depending on MACS status in cardiovascular risk profile with the sole exception being a 3-fold elevation in CVD prevalence in those with MACS (40.8 vs. 13.3%, *p* < 0.05) (Table S3). Descriptive data on antidiabetic drug use in those with T2D, stratified according to MACS status, are shown in *Table S4*. Of the patients with T2D, those with MACS (Table S3) had a trend towards more insulin use (22.4 vs. 10.7%, *p* = 0.09, *Table S4*) and slightly higher HbA1c levels (6.9 vs. 6.6%, *p* = 0.04, *Figure **S1*) compared to those with T2D and NFAA (Table S3).

**Table 1:** Baseline characteristics of patients with and without T2D. T2D - type 2 diabetes mellitus, BMI - body mass index, DST - dexamethasone suppression test, CVD - cardiovascular disease, aHTN - arterial hypertension, ^§^ Wilcoxon rank-sum test, ^$^ chi^2^ test of independence

variable	Metric	T2D	No T2D	*p*-value
Age [years]	**Median (IQR)**	63 (11)	58 (16)	< 0.001 ^§^
Men	**n (%)**	68/151 (45.0%)	178/517 (34.4%)	0.026 ^$^
Women	**n (%)**	83/151 (55.0%)	339/517 (65.6%)	
BMI [kg/m2]	**Median (IQR)**	29.7 (8.2)	27.2 (6.5)	< 0.001 ^§^
Post 1 mg DST cortisol [µg/dL]	**Median (IQR)**	1.9 (1.9)	1.5 (1.5)	0.006 ^§^
aHTN	**n (%)**	129/151 (85.4%)	316/517 (61.1%)	< 0.001 ^$^
Dyslipidemia	**n (%)**	95/151 (62.9%)	178/517 (34.4%)	< 0.001 ^$^
CVD	**n (%)**	41/151 (27.2%)	42/517 (8.1%)	< 0.001 ^$^
Osteoporosis	**n (%)**	4/151 (2.7%)	27/517 (5.2%)	0.270 ^$^

### Mortality analysis

Over a retrospective observational period of up to 24.9 years (median follow-up time 9.0 years), there were a total of 96 documented deaths. Causes of death according to hypercortisolism state are descriptively listed in Table S5. The median and maximum follow-up times per group can be obtained from Table S6.

**Fig. 2:**
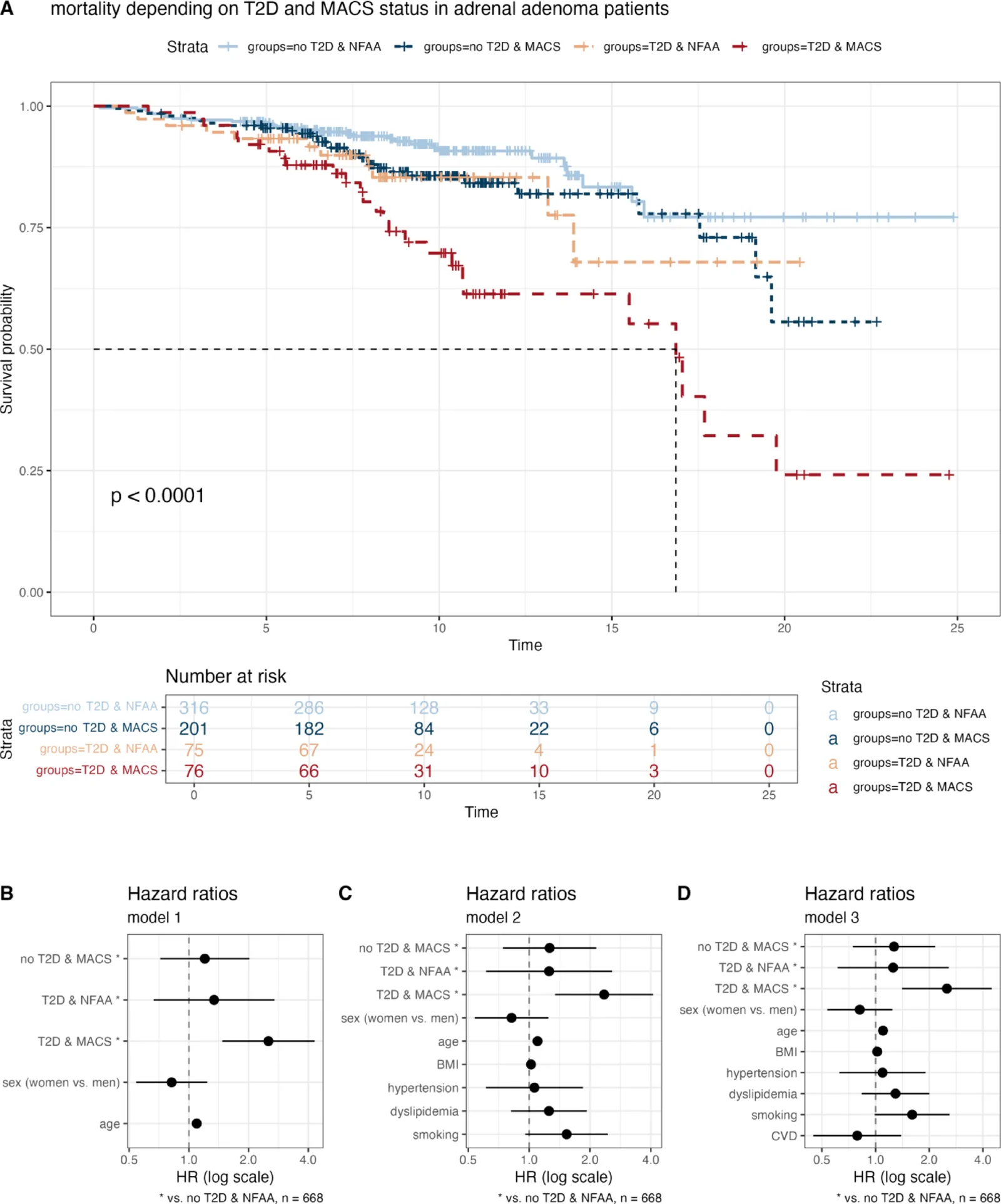
**A** Kaplan-Meier survival analysis according to T2D and MACS status. **B** Cox proportional hazards model comparing the 3 exposure groups to the double-negative control cohort, adjusted for sex and age, as well as **C** BMI, hypertension, dyslipidemia and smoking and **D** CVD. T2D - type 2 diabetes mellitus, MACS - mild autonomous cortisol section, BMI - body mass index, CVD - cardiovascular disease, HR - hazard ratio

In the overall population, Kaplan–Meier survival distributions differed significantly across the four groups (Fig. 2A). The 10-year survival rate was 0.7 [CI95%: 0.59–0.83] in the T2D & MACS cohort – compared to this, survival rates were higher in the T2D & NFAA cohort (0.85 [CI95%: 0.77–0.95]), as well as the no T2D & MACS cohort (0.86 [CI95%: 0.80–0.91]) and the no T2D & NFAA cohort (0.91 [CI95%: 0.87–0.95]) (Table S7). We then fitted cox proportional hazards models to account for potential confounding factors in a stepwise manner. Compared to the reference cohort (no T2D & NFAA) patients in the T2D & MACS cohort had significantly increased mortality (HR 2.52 [CI95%: 1.47–4.30], *p* < 0.001) after adjusting for age and sex (Fig. 2B, Table S8). This relationship persisted even after successive model building with inclusion of additional cardiometabolic covariates, including BMI, arterial hypertension, dyslipidemia, smoking (T2D & MACS: HR 2.35 [CI95% 1.34–4.10], *p* < 0.01, Fig. 2C, *Table S8*) and CVD at baseline (T2D & MACS: HR 2.51 [CI95%: 1.41–4.48], *p* < 0.01, Fig. 2D, Table S8). These results did not change in sensitivity analyses where all patients with baseline CVD were excluded (T2D & MACS: HR 2.38 [CI95% 1.24–4.55], *p* < 0.05, Table S9) or when accounting for adrenalectomy (T2D & MACS: HR 2.65 [CI95%1.47–4.77], *p* < 0.01, Table S10).

To further test the interplay of T2D and MACS status, we fitted cox proportional hazards models based on full covariate accounting as in model 3 (Fig. 2D) where we assessed models with and without a T2D × MACS interaction term. In the main effects analysis, T2D, but not MACS status, was significantly associated with increased mortality (T2D: HR 1.65 [CI95% 1.05–2.58], *p* < 0.05, MACS: HR 1.49 [CI95% 0.97–2.29], *p* = 0.07). A direct interaction term (T2D:MACS) was not significantly associated with mortality (Table S11).

## Discussion

This study highlights the increased mortality of patients with type 2 diabetes mellitus in the context of adrenal hypercortisolism. Compared to patients with non-functioning adrenal adenomas without T2D, MACS was strongly linked to increased mortality only in T2D but not in those without T2D, even after adjustment for age, sex, BMI, smoking, hypertension, dyslipidemia and CVD at baseline. These findings suggest that the excess mortality observed in patients with MACS and T2D may reflect effect modification by T2D and potentially a more severe metabolic phenotype with the association persisting after adjustment for baseline CVD.

The link between increased mortality due to even mild disruptions of the hypothalamic-pituitary-adrenal axis has been repeatedly reported [2, 20, 21]. Deutschbein et al. reported higher all-cause mortality in patients with autonomous cortisol secretion and Di Dalmazi and Patrova et al. found mortality to be associated with post 1 mg DST cortisol concentrations in the general population with adrenal incidentalomas [2, 20, 21]. In addition, the systematic review by Pelsma et al. confirmed an association of mild autonomous cortisol secretion with increased mortality and adverse cardiometabolic outcomes [6]. In the study at hand, we underscore this notion seen in the general population. However, we are the first to show that especially T2D can dramatically increase the mortality rate in patients with MACS. Importantly, increased mortality in patients with MACS and T2D remained significant after adjustment for age, sex, BMI, smoking, hypertension, dyslipidemia and CVD at baseline as well as for adrenalectomy. These findings suggest that presence of T2D may identify a clinically vulnerable phenotype in which even mild autonomous cortisol secretion might lead to a more severe T2D phenotype, translating into adverse survival outcomes – like development of CVD over the decades. Use of GLP-1 receptor agonists and SGLT2 inhibitors was low in both T2D subgroups, likely reflecting the early recruitment period. Thus, whether their known cardiovascular benefits [22–25] could improve outcomes particularly in T2D patients with adrenal hypercortisolism remains to be explored.

In the literature, an association between hypercortisolism and cardiovascular disease has been described repeatedly, with cardiovascular disease mostly representing the leading cause of death [6, 10, 20]. Glucocorticoid excess adversely affects several key metabolic pathways, including glucose metabolism [3, 4]. In fact, a recent meta-analysis reported that up to 14% of patients with diabetes mellitus present with an abnormal DST [7]. These cortisol-related alterations may contribute to cardiovascular morbidity and mortality, particularly in metabolically vulnerable patients. Patients with T2D and MACS may have been exposed to a “double metabolic stress” of established diabetes and chronic mild cortisol excess. Particularly, under earlier guidelines patients with post DST cortisol > 1.8–5.0 µg/dL may have remained under-recognized despite increased risk. Concomitant T2D and cortisol excess may have further impaired glycemic control and contributed to adverse outcomes. Consistently, Prete et al. reported a higher prevalence of insulin-treated diabetes among patients with adrenal hypercortisolism [26], while Chen et al. found fasting and postprandial hyperglycemia and lower postprandial insulin secretion in patients with MACS [15]. Supporting these findings, we observed significantly higher HbA1c levels in patients with T2D and MACS compared to T2D patients with NFAA. Conversely, poor glycemic control and chronic low-grade systemic inflammation [27] may itself contribute to non-neoplastic hypercortisolism (pseudo-Cushing) and impaired cortisol suppression following DST, potentially confounding the observed association [28]. Thus, reverse causation cannot be excluded. Nevertheless, the observed increased mortality in patients with T2D and MACS in our study population supports the clinical relevance of their combined presence.

The DST may therefore help to unmask patients with a more severe metabolic systemic inflammatory phenotype, thus potentially identifying patients with T2D at increased mortality risk.

The absence of an effect on mortality in patients without T2D in our cohort may partly be explained by the higher BMI observed in the NFAA group, which could have attenuated the apparent cardiovascular benefit associated with lower cortisol exposure. Notably, the unexpectedly low osteoporosis rate in patients with MACS and T2D may partly be explained by the tendency of Dual-energy X-ray absorptiometry to overestimate bone mineral density in patients with T2D [29, 30].

Nevertheless, these findings support further diagnostic evaluation and careful consideration of treatment strategies in patients with diabetes and adrenal adenomas. Several studies have reported improvements in metabolic parameters following treatment of cortisol excess. Yu et al. described resolution or improvement of diabetes after unilateral adrenalectomy for ACTH-independent Cushing’s syndrome [31]. Morelli et al. reported that adrenalectomy improved blood pressure and glycemic control compared with conservative management in patients with adrenal incidentaloma and post 1 mg DST non-suppression, while Koh et al. found that adrenalectomy improved weight, glucose, and blood pressure control in patients with MACS [32, 33]. Also, improvement of ectopic lipid profiles, insulin sensitivity and blood pressure under metyrapone treatment in patients with MACS has been found [34, 35].

Of note, due to the retrospective character of the present study, recall bias leading to omissions of comorbidities is a possible limitation. Likewise, we had no information about possible influencing factors such as data on acute mental stress, alcohol use, depression, renal and hepatic dysfunction. Furthermore, the limited consideration of treatment strategies in the present study represents an additional limitation. In addition, as most cases of endogenous Cushing’s syndrome are of pituitary origin, the present findings are specifically limited to adrenal cortisol excess and may not be generalizable to other forms of endogenous hypercortisolism. Another limitation is the absence of an adenoma-free control group, as NFAA have also been associated with an increased risk of diabetes [36]. Also, prediabetes was not separately analyzed in this study. Finally, given the long follow-up period, changes in T2D status and cortisol secretion over time were not accounted for, potentially resulting in time-dependent misclassification bias, although progression from MACS to overt Cushing’s syndrome is considered exceedingly rare [1, 37]. Further confirmatory studies incorporating larger sample sizes and including an adenoma-free control group ought to be conducted on this matter.

## Conclusions

In the present study, we show that among patients with adrenal adenomas, MACS is related to an increased mortality rate in patients with T2D. Together, these observations suggest that patients with T2D may represent a subgroup in whom even mild forms of adrenal hypercortisolism are of particular metabolic and prognostic relevance. Our findings therefore provide a rationale for prospective studies focusing on patients with T2D and adrenal adenomas to further characterize cardiometabolic risk profiles in the context of MACS to determine whether targeted treatment of hypercortisolism can improve not only metabolic parameters, but also cardiovascular and survival outcomes.

## Supplementary Information

Below is the link to the electronic supplementary material.

Supplementary Material 1.

## Data Availability

The datasets used and analysed during the current study are available from the corresponding author on reasonable request.

## Abbreviations

MACS: Mild autonomous cortisol secretion
NFAA: Non-functioning adrenal adenoma
ESE: European Society of Endocrinology
ADA: American diabetes association
DST: Dexamethasone suppression test
CVD: Cardiovascular disease
T2D: Type 2 diabetes mellitus
aHTN: Arterial hypertension
BMI: Body mass index
