# Supplementary material for: Mild autonomous cortisol secretion (MACS) is associated with increased mortality in patients with adrenal adenomas only in the presence of type 2 diabetes: a retrospective cohort study
Source: Cardiovasc Diabetol. 2026 Sep 22;25:294. doi: 10.1186/s12933-026-03360-4 (PMC13621561; doi:10.1186/s12933-026-03360-4)
Supplement: Supplementary file 1 [file 12933_2026_3360_MOESM1_ESM.docx]

**Mild autonomous cortisol secretion (MACS) is associated with increased mortality in patients with adrenal adenomas only in the presence of type 2 diabetes – a retrospective cohort study**

Luise Bellach ^1*^, Sarah Hofer-Zeni^1*^, Dorota Sluková ^1^, Dorothea Kalender ^1^, Clemens Zimmermann ^1^, Naima Michler ^1^, Liliana Gard ^1^, Sabina Baumgartner-Parzer ^1^, Rodrig Marculescu ^2^, Greisa Vila ^1^, Peter Wolf ^1^, Alexandra Kautzky-Willer ^1^, Michael Leutner ^1^

^*^ contributed equally

^1^ Department of Medicine III, Division of Endocrinology and Metabolism, Medical University of Vienna, Vienna, Austria

^2^ Department of Laboratory Medicine, Medical University of Vienna, Vienna 1090, Austria

**Supplements**

| names | metric | MACS | NFAA | p-value |
| --- | --- | --- | --- | --- |
| ACTH [pg/mL] | Median (IQR) | 7 (8) | 13 (10) | 0.001 |
| DHEAS [µg/mL] | Median (IQR) | 0.53 (0.6) | 0.85 (1.0) | <0.001 |

*Table S1 : comparison of baseline ACTH and DHEAS levels between those with MACS and NFAA.*

| Drug Class | T2D | No T2D |
| --- | --- | --- |
| ACEi | 57/151 (37.7%) | 109/517 (21.1%) |
| ARB | 61/151 (40.4%) | 157/517 (30.4%) |
| BB | 6/151 (4%) | 21/517 (4.1%) |
| CCB | 56/151 (37.1%) | 125/517 (24.2%) |
| alpha-blockers | 2/151 (1.3%) | 11/517 (2.1%) |
| DDP4i | 36/151 (23.8%) | 0/517 (0%) |
| GLP1A | 2/151 (1.3%) | 0/517 (0%) |
| SGLT2i | 6/151 (4%) | 0/517 (0%) |
| acarbose | 3/151 (2%) | 1/517 (0.2%) |
| glitazones | 5/151 (3.3%) | 0/517 (0%) |
| insulin | 25/151 (16.6%) | 0/517 (0%) |
| metformin | 92/151 (60.9%) | 1/517 (0.2%) |
| ezetimibe | 7/151 (4.6%) | 9/517 (1.7%) |
| fibrates | 4/151 (2.6%) | 10/517 (1.9%) |
| statins | 79/151 (52.3%) | 95/517 (18.4%) |

Table S2: distribution of drugs in patients with and without T2D. ACEi … angiotensin converting enzyme inhibitor, ARB … angiotensin II receptor blockers, BB … beta blockers, CCB … calcium channel blockers, DPP4i … dipeptidyl peptidase-4 inhibitor, GLP1A … glucagon-like-peptide 1 receptor agonists, SGLT2i … sodium-glucose transporters 2 inhibitors; one patient in the non-T2D group was taking acarbose for symptomatic treatment of dumping symptoms and another patient in the non-T2D group was taking metformin due to polycystic ovary syndrome.

| variable | metric | No T2D & NFAA | No T2D & MACS | T2D & NFAA | T2D & MACS | p-value |
| --- | --- | --- | --- | --- | --- | --- |
| Age [years] | **median (IQR)** | 56 (15.2) ^a,b,c^ | 60 (15) ^a^ | 62 (9.5) ^b^ | 63 (12.2) ^c^ | <0.001 ^§^ |
| Men | **n (%)** | 118/316 (37.3%) | 60/201 (29.85%) | 34/75 (45.3%) | 34/76 (44.7%) | 0.035 ^$^ |
| Women | **n (%)** | 198/316 (62.7%) | 141/201 (70.15%) | 41/75 (54.7%) | 42/76 (55.3%) |  |
| BMI [kg/m2] | **median (IQR)** | 28.2 (7) ^a,b^ | 26.2 (6) ^a,d,e^ | 30.5 (8.2) ^b,d^ | 29.3 (7.8) ^e^ | <0.001 ^§^ |
| Post 1mg DST cortisol [µg/dL] | **median (IQR)** | 1.1 (0.5) ^a,c^ | 2.9 (3.3) ^a,d^ | 1.2 (0.6) ^d,f^ | 3.1 (3) ^c,f^ | <0.001 ^§^ |
| aHTN | **n (%)** | 182/316 (57.6%) ^b,c^ | 134/201 (66.7%) ^d,e^ | 62/75 (82.7%) ^b,d^ | 67/76 (88.2%) ^c,e^ | <0.001 ^$^ |
| dyslipidemia | **n (%)** | 109/316 (34.5%) ^b,c^ | 69/201 (34.3%) ^d,e^ | 51/75 (68.0%) ^b,d^ | 44/76 (57.9%) ^c,e^ | <0.001 ^$^ |
| CVD | **n (%)** | 22/316 (7.0%) ^c^ | 20/201 (10.0%) ^e^ | 10/75 (13.3%) ^f^ | 31/76 (40.8%) ^c,e,f^ | <0.001 ^$^ |
| osteoporosis | **n (%)** | 8/316 (2.5%) ^a^ | 19/201 (9.5%) ^a^ | 2/75 (2.7%) | 2/76 (2.6%) | 0.004 ^&^ |

Table S3: Baseline characteristics of patients with and without T2D, according to MACS status. T2D … type 2 diabetes mellitus, MACS … mild autonomous cortisol secretion, BMI … body mass index, DST … dexamethasone suppression test, CVD … cardiovascular disease, aHTN … arterial hypertension, ^§^ … Kruskal Wallis test, ^$^ … chi^2^ test of independence, ^&^ … Fisher's exact test

^a^ … adjusted p < 0.05 for No T2D & NFAA vs. no T2D & MACS

^b^ … adjusted p < 0.05 for No T2D & NFAA vs. T2D & NFAA

^c^ … adjusted p < 0.05 for No T2D & NFAA vs. T2D & MACS

^d^ … adjusted p < 0.05 for No T2D & MACS vs. T2D & NFAA

^e^ … adjusted p < 0.05 for No T2D & MACS vs. T2D & MACS

^f^ … adjusted p < 0.05 for T2D & NFAA vs. T2D & MACS

| **drug class** | **NFAA** | **MACS** |
| --- | --- | --- |
| DDP4i | 22/75 (29.3%) | 14/76 (18.4%) |
| GLP1A | 1/75 (1.3%) | 1/76 (1.3%) |
| SGLT2i | 5/75 (6.7%) | 1/76 (1.3%) |
| acarbose | 1/75 (1.3%) | 2/76 (2.6%) |
| insulin | 8/75 (10.7%) | 17/76 (22.4%) |
| metformin | 48/75 (64%) | 44/76 (57.9%) |
| glitazones | 5/75 (6.7%) | 0/76 (0%) |

Table S4: distribution of diabetes therapies among patients with T2D, stratified according to MACS status

Figure S1: HbA1c according to T2D and MACS status

| Cause of death | NFAA | MACS |
| --- | --- | --- |
| CVD | 13 | 16 |
| cancer | 12 | 19 |
| gastrointestinal | 2 | 3 |
| hematologic | 2 | 2 |
| infectious | 1 | 1 |
| metabolic, endocrine | 1 | 4 |
| neurologic, psychiatric | 3 | 3 |
| other | 4 | 1 |
| renal failure | 2 | 0 |
| pulmonary | 0 | 7 |

Table S5: Causes of death in deceased patients (n = 96). CVD … cardiovascular disease

| groups | Min. | 1st Qu. | Median | Mean | 3rd Qu. | Max. | PY | unit |
| --- | --- | --- | --- | --- | --- | --- | --- | --- |
| no T2D & NFAA | 0.2 | 6.8 | 9.2 | 9.6 | 11.4 | 24.9 | 3035.1 | years |
| no T2D & MACS | 0.5 | 6.9 | 9.5 | 9.6 | 11.2 | 22.7 | 1933.9 |  |
| T2D & NFAA | 0.9 | 6.1 | 8.1 | 8.7 | 10.9 | 20.4 | 649.9 |  |
| T2D & MACS | 1.6 | 6.3 | 8.3 | 9.3 | 10.8 | 24.8 | 705.7 |  |

Table S6: survival rates over 20 years, split according to T2D and MACS status. Min … minimum, Qu … quantile, Max … maximum, PY … total person years

| strata | time | n.risk | n.event | n.censor | surv | CI2.5% | CI97.5% |
| --- | --- | --- | --- | --- | --- | --- | --- |
| no T2D & NFAA | 10 | 128 | 23 | 165 | 0.91 | 0.87 | 0.95 |
|  | 15 | 33 | 4 | 91 | 0.83 | 0.76 | 0.92 |
|  | 20 | 9 | 2 | 22 | 0.77 | 0.67 | 0.89 |
|  |  |  |  |  |  |  |  |
| no T2D & MACS | 10 | 84 | 24 | 93 | 0.86 | 0.80 | 0.91 |
|  | 15 | 22 | 2 | 60 | 0.82 | 0.75 | 0.90 |
|  | 20 | 6 | 4 | 12 | 0.56 | 0.36 | 0.85 |
|  |  |  |  |  |  |  |  |
| T2D & NFAA | 10 | 24 | 9 | 42 | 0.85 | 0.77 | 0.95 |
|  | 15 | 4 | 2 | 18 | 0.68 | 0.48 | 0.95 |
|  | 20 | 1 | 0 | 3 | 0.68 | 0.48 | 0.95 |
|  |  |  |  |  |  |  |  |
| T2D & MACS | 10 | 31 | 18 | 27 | 0.70 | 0.59 | 0.83 |
|  | 15 | 10 | 3 | 18 | 0.61 | 0.49 | 0.77 |
|  | 20 | 3 | 5 | 2 | 0.24 | 0.10 | 0.59 |

Table S7: survival rates over 20 years, split according to T2D and MACS status

| model 1 | | | | |
| --- | --- | --- | --- | --- |
| term | **HR** | **CI2.5%** | **CI97.5%** | **p-value** |
| groups: no T2D & MACS * | 1.20 | 0.72 | 2.02 | 0.483 |
| groups: T2D & NFAA * | 1.34 | 0.67 | 2.70 | 0.413 |
| groups: T2D & MACS * | 2.52 | 1.47 | 4.30 | <0.001 |
| sex: women | 0.82 | 0.54 | 1.24 | 0.341 |
| age | 1.10 | 1.07 | 1.12 | <0.001 |
| model 2 | | | | |
| term | **HR** | **CI2.5%** | **CI97.5%** | **p-value** |
| groups: no T2D & MACS * | 1.26 | 0.74 | 2.15 | 0.386 |
| groups: T2D & NFAA * | 1.25 | 0.61 | 2.57 | 0.531 |
| groups: T2D & MACS * | 2.35 | 1.34 | 4.10 | 0.003 |
| sex: women | 0.82 | 0.54 | 1.25 | 0.346 |
| age | 1.10 | 1.07 | 1.13 | <0.001 |
| BMI | 1.02 | 0.99 | 1.06 | 0.208 |
| aHTN | 1.06 | 0.61 | 1.84 | 0.829 |
| dyslipidemia | 1.25 | 0.81 | 1.93 | 0.302 |
| smoking | 1.53 | 0.96 | 2.45 | 0.074 |
| model 3 | | | | |
| term | **HR** | **CI2.5%** | **CI97.5%** | **p-value** |
| groups: no T2D & MACS * | 1.27 | 0.75 | 2.16 | 0.376 |
| groups: T2D & NFAA * | 1.25 | 0.61 | 2.57 | 0.532 |
| groups: T2D & MACS * | 2.51 | 1.41 | 4.48 | 0.002 |
| sex: women | 0.82 | 0.54 | 1.24 | 0.337 |
| age | 1.10 | 1.07 | 1.13 | <0.001 |
| BMI | 1.02 | 0.99 | 1.06 | 0.214 |
| aHTN | 1.09 | 0.63 | 1.91 | 0.748 |
| dyslipidemia | 1.29 | 0.84 | 2.00 | 0.245 |
| smoking | 1.60 | 0.99 | 2.60 | 0.055 |
| CVD | 0.79 | 0.45 | 1.39 | 0.408 |

Table S8: model output of models featured in Figure 2B-D. * compared to the reference group (no T2D & NFAA).

| term | HR | CI2.5% | CI97.5% | p-value |
| --- | --- | --- | --- | --- |
| groups: no T2D & MACS * | 0.95 | 0.53 | 1.70 | 0.850 |
| groups: T2D & NFAA * | 1.13 | 0.54 | 2.40 | 0.740 |
| groups: T2D & MACS * | 2.38 | 1.24 | 4.55 | 0.010 |
| sex: women | 0.76 | 0.47 | 1.22 | 0.248 |
| age | 1.11 | 1.08 | 1.14 | <0.001 |
| BMI | 1.01 | 0.97 | 1.05 | 0.524 |
| aHTN | 1.30 | 0.70 | 2.40 | 0.394 |
| dyslipidemia | 1.34 | 0.83 | 2.17 | 0.230 |
| smoking | 1.81 | 1.02 | 3.23 | 0.044 |

Table S9: sensitivity analysis of model 2 (Figure 2C), all patients with baseline CVD excluded. * compared to the reference group (no T2D & NFAA).

| term | HR | CI2.5% | CI97.5% | p-value |
| --- | --- | --- | --- | --- |
| groups: no T2D & MACS * | 1.31 | 0.77 | 2.23 | 0.322 |
| groups: T2D & NFAA * | 1.26 | 0.62 | 2.59 | 0.521 |
| groups: T2D & MACS * | 2.65 | 1.47 | 4.77 | 0.002 |
| sex: women | 0.83 | 0.55 | 1.27 | 0.391 |
| age | 1.10 | 1.07 | 1.13 | <0.001 |
| BMI | 1.02 | 0.99 | 1.06 | 0.228 |
| aHTN | 1.10 | 0.63 | 1.93 | 0.724 |
| dyslipidemia | 1.29 | 0.83 | 1.99 | 0.253 |
| smoking | 1.63 | 1.00 | 2.64 | 0.048 |
| CVD | 0.78 | 0.44 | 1.37 | 0.380 |
| ADX | 0.67 | 0.26 | 1.73 | 0.402 |

Table S10: sensitivity analysis of model 3 (Figure 2D), also accounting for adrenalectomy. * compared to the reference group (no T2D & NFAA). ADX … adrenalectomy. Adrenalectomy rates per group: no T2D & NFAA: n = 9, no T2D & MACS: n = 29, T2D & NFAA: n = 2, T2D & MACS: n = 13

| model 4: main effects | | | | |
| --- | --- | --- | --- | --- |
| term | **HR** | **CI2.5%** | **CI97.5%** | **p-value** |
| T2D | 1.65 | 1.05 | 2.58 | 0.031 |
| MACS | 1.49 | 0.97 | 2.29 | 0.069 |
| Sex (women) | 0.81 | 0.53 | 1.24 | 0.326 |
| age | 1.10 | 1.07 | 1.13 | <0.001 |
| BMI | 1.02 | 0.99 | 1.06 | 0.211 |
| aHTN | 1.10 | 0.63 | 1.91 | 0.736 |
| dyslipidemia | 1.30 | 0.84 | 2.01 | 0.235 |
| smoking | 1.59 | 0.98 | 2.57 | 0.060 |
| CVD | 0.83 | 0.47 | 1.44 | 0.502 |
| model 5: T2D x MACS interaction | | | | |
| term | **HR** | **CI2.5%** | **CI97.5%** | **p-value** |
| T2D | 1.25 | 0.61 | 2.57 | 0.532 |
| MACS | 1.27 | 0.75 | 2.16 | 0.376 |
| Sex (women) | 0.82 | 0.54 | 1.24 | 0.337 |
| age | 1.10 | 1.07 | 1.13 | <0.001 |
| BMI | 1.02 | 0.99 | 1.06 | 0.214 |
| aHTN | 1.09 | 0.63 | 1.91 | 0.748 |
| dyslipidemia | 1.29 | 0.84 | 2.00 | 0.245 |
| smoking | 1.60 | 0.99 | 2.60 | 0.055 |
| CVD | 0.79 | 0.45 | 1.39 | 0.408 |
| T2D:MACS | 1.58 | 0.64 | 3.90 | 0.318 |

Table S11: main effects (model 4) and interaction effects (model 5) models.
